# Nesting strategy reflects individual worker movement in termites

**DOI:** 10.1186/s40462-025-00612-y

**Published:** 2025-12-29

**Authors:** Kensei Kikuchi, Thomas Bourguignon, Nobuaki Mizumoto

**Affiliations:** 1 Okinawa Institute of Science & Technology Graduate University, Onna-son, Okinawa, 904-0495 Japan; 2 Department of Entomology & Plant Pathology, Auburn University, Auburn, AL 36849 USA

**Keywords:** Nest, Phylogenetic comparative analysis, Social evolution

## Abstract

Termite nests widely vary in size and complexity across species. While nest structure reflects the social organisation of termite colonies, its impact on individual-level behaviour is unknown. Here, we show that termite worker movements are strongly correlated with their nesting strategies. We compared one-piece nesters, whose colony life cycle is completed within a single piece of wood serving as nest and food, to foragers, which make colonies exploiting resources spanning several wood items. Our observations of worker movements revealed that one-piece nesters moved significantly less than foragers. The distributions of movement duration were also different, with foragers showing a tendency to sustain locomotion once initiated, while the locomotion of one-piece nesters stopped within a short period. On the contrary, the distributions of pausing duration were broadly consistent across species, with a notable exception in *C. formosanus.* Spatial analyses further showed that foragers exhibited stronger wall-following behavior and traversed the arena center more straightly than one-piece nesters. Thus, nesting strategies are reflected not only in movement activities but also in the spatial exploration of novel environments. These findings provide a new framework for understanding how nesting strategies drive the evolution of movement patterns across social species.

**Supplementary Information:**

The online version contains supplementary material available at 10.1186/s40462-025-00612-y.

## Introduction

Many social insects, including ants, bees, termites, and wasps, build nests in which they live. The nest configuration varies among species, ranging from simple soil cavities to sprawling nests with intricate networks of tunnels [1–3]. One essential function of social insect nests is to provide a safe and stable environment. In addition, nest structure dictates the movement patterns of colony members, modulating spatial and temporal patterns of behavioural interactions among colony members [4–6]. For example, in harvester ant (*Veromessor andrei*) nests, entrance chambers form highly interconnected corridors that enable rapid pheromone diffusion and simultaneous worker access, markedly accelerating foraging recruitment [7]. Similarly, the hierarchical gallery networks built by *Cubitermes* termites minimize path lengths and traffic congestion—thereby maximizing transport efficiency—while also creating defensive choke points against intruders [8]. Because these nesting architectures are often species-specific, the consistent tendency of individuals to traverse particular nest regions may create an innate movement bias between different species. Although previous studies revealed the behavioural mechanisms that produce diverse nest structures [9, 10], how nest structures shape social insect behaviour remains unknown.

**Fig. 1:**
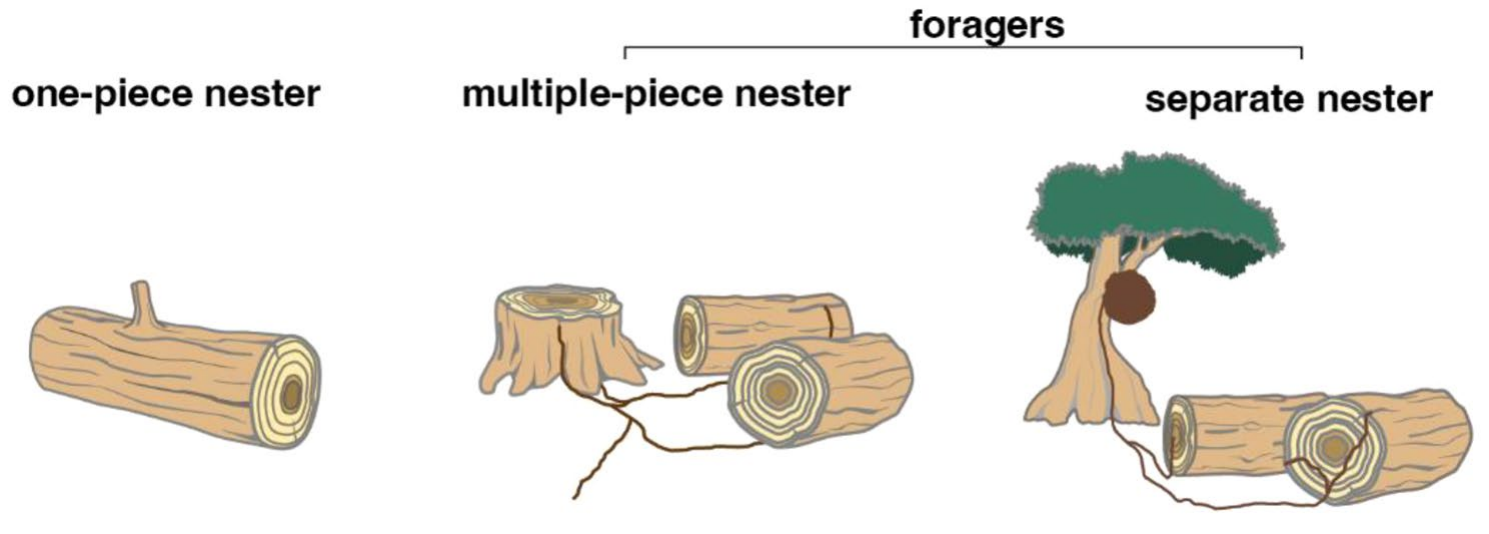
Nesting types of termites. One-piece nesters are enclosed within a single piece of wood serving both as nest and food source. Multiple-piece nesters feed on and nest in separate wood items interconnected by underground tunnels or aboveground shelter tubes. Separate nesters build nests separated from food resources

Termites provide a compelling model for studying the relationship between nest organisation and social behaviour. Termite nest organisations are generally classified into three types [11]: one-piece nesters, multiple-piece nesters, and separate nesters (Fig. 1). One-piece nesters reside in a single piece of wood that functions as both shelter and food. Their life cycles are completed within their nests, which they do not leave to forage. In contrast, multiple-piece and separate nesters, termed here foragers [12, 13], come out of their nests to exploit multiple food sources. Multiple-piece nesters live in colonies spreading across multiple pieces of wood connected by underground tunnels or aboveground shelter tubes, while separate nesters build and live in nests separated from the food resources they exploit (e.g., subterranean nests, mounds at the soil surface, and arboreal nests). The nesting type entails substantial differences in living areas. While multiple-piece nesters and separate nesters can forage over distances of 100 m [14, 15], one-piece nesters do not forage, and their nest is contained within a single piece of wood. We posit that these life-history differences affect movement patterns, especially the movements of workers, which are responsible for foraging and feeding.

In this study, we investigated the relationship between nesting types and worker movement patterns in five termite species: the one-piece nesters *Neotermes sugioi* and *Zootermopsis nevadensis*, and the foragers *Hodotermopsis sjostedti*, *Coptotermes formosanus,* and *Odontotermes formosanus*. Note that *H. sjostedti* was often treated as a one-piece nester until they were reclassified as a multiple-piece nester [16], *C. formosanus* often shows intermediate nesting between multiple-piece and separate nesters [17], and *O. formosanus* is clearly a separate nester that establishes fungus-growing gardens. We quantitatively and qualitatively compared the movement patterns of isolated termite workers. We first compared the distance traveled by workers of the five species to test the link between nesting strategies and movement activities. Second, we characterised the movement patterns of each termite species by fitting probabilistic models of animal movements to the duration of movement and pausing behaviour [18–21]. Finally, we compared the time spent in the inner/outer areas of the arena between one-piece nesters and foragers. Previous studies showed that foragers typically follow the wall [19, 22, 23], but it is unclear whether one-piece nesters follow the same movement patterns. By comparing termite species with divergent nesting strategies in a controlled context, we isolated the effect of life history and nest architecture on movements. This approach allows us to uncover innate behavioral patterns potentially shaped by long-term evolutionary pressures.

## Materials and methods

### Sampling

We collected colonised pieces of wood for five species of termites in Japan. We collected *N. sugioi* in Okinawa Island, Okinawa, *Z. nevadensis* in Kawanishi, Osaka, *C. formosanus* in Okinawa Island, Okinawa, *H. sjostedti* in Amami Island, Kagoshima, and *O. formosanus* in Naha, Okinawa. We collected five colonies for each species. Colonies of multiple-piece and separate nesters were collected in locations distant of at least 200 m. We brought all colonies back to the lab and maintained them at 20 °C until the experiments.

### Behavioural observations

We observed the movements of one individual worker or pseudergate at a time in an experimental arena consisting of a glass Petri dish (Ø = 145 mm) lined with wet filter paper (Ø = 140 mm). *C. formosanus* and *O. formosanus* have a true worker caste composed of individuals that irreversibly deviated from the imaginal developmental line at an early developmental stage and cannot moult into alate imagoes [12]. On the contrary, *N. sugioi*, *Z. nevadensis*, and *H. sjostedti* lack a true worker caste, and colony tasks are performed by immatures, called pseudergates, which retain the potential to develop into alate imagoes [12]. In this study, we refer to both true workers and pseudergates as workers. We recorded the behaviour of five workers from each colony. Each worker was used only once for data collection. Before each trial, we inspected each worker to ensure that all antennae and legs were intact and that no external parasites (e.g., mites) were present. Individuals were then transferred one at a time into the arena using an aspirator. Each termite was gently released in the center of the Petri dish from a height of less than 3 cm (i.e., not physically dropped) and allowed to acclimate for 15 min. Recording only began after the individual exhibited normal walking behaviour.

Arenas were placed in a white acrylic chamber (200 mm × 180 mm x 180 mm), surrounded by LED tape light to render enough contrast between termites and the background (300–500 lux). We covered acrylic chambers with blackout curtains to keep lighting conditions constant during trials. A Raspberry Pi Camera Module (Raspberry Pi NoIR Camera V2) connected to a single-board computer (Raspberry Pi 4 model B) was mounted on top of the experimental set-up. Videos were recorded using a web interface, RPi-Cam-Web-Interface (https://elinux.org/RPi-Cam-Web-Interface), at 25 frames per second for 60 minutes and a resolution of 1296 × 972 pixels. Before each trial, we thoroughly cleaned the surface of the petri dish with 70% ethanol and renewed the filter paper. No termites died during the experiments.

### Movement analysis

We obtained the trajectories of each termite worker from each video clip using the animal-tracking software UMATracker [24]. Coordinates were recorded in pixels and converted into mm using a scale bar (30 ×10 mm) placed inside the experimental chamber as a reference. We downsampled the data to 5 frames per second for the trajectory analysis.

**Fig. 2:**
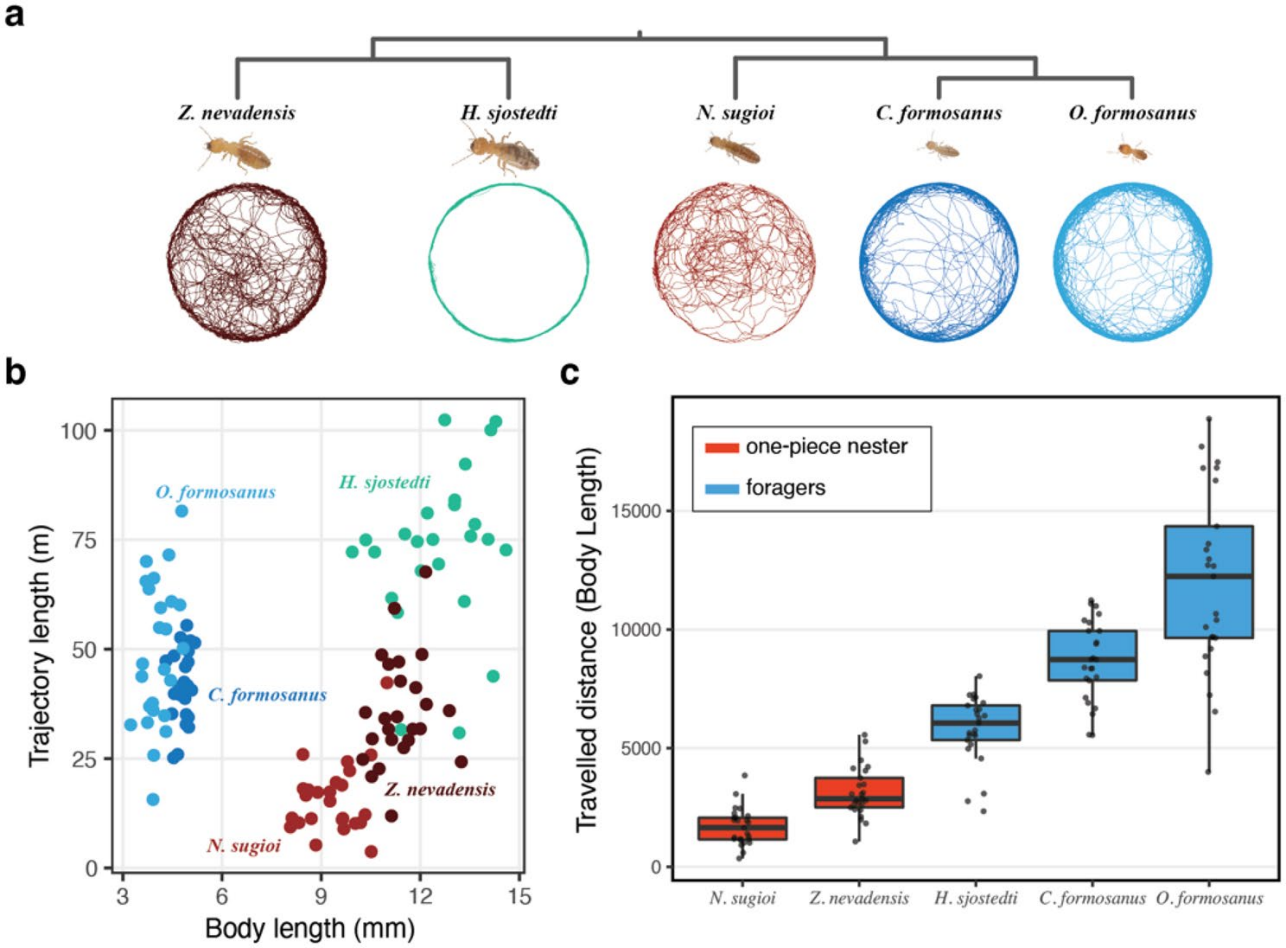
Comparison of moving activities across termite species. (**a**) cladogram showing the phylogenetic relationships between the five studied termite species, with representative movement patterns. (**b**) comparison of body size and traveled distance. (**c**) comparison of traveled distances standardised by body length. Foraging species traveled significantly longer distances than one-piece nesting species (LMM, Type-II Wald χ^2^ test, χ^2^ = 7.51, *p* = 0.0061; *n* = 50 one-piece, *n* = 75 foragers)

We used the distances traveled by each individual between two successive frames (step length) to compare the traveled distances and the movement and pausing patterns among termite species. By summing up all step lengths for each individual, we obtained the total traveled distance for the recorded 60 minutes. We also measured the body length of each individual to standardise traveled distances. Note that there was no significant correlation between body size and traveled distances within species (Fig. 2B; correlation coefficient *r* = 0.106 for *C. formosanus*; *r* = 0.146 for *H. sjostedti*; *r* = 0.199 for *N. sugioi*; *r* = 0.210 for *O. formosanus*; *r* = 0.105 for *Z. nevadensis*). To test for differences between one-piece nesters and foragers, we used linear mixed models (LMMs) with nesting type as a fixed effect and colony identity nested within species as a random intercept (1|Species/Colony). Models were fitted using the lmer() function in the lme4 package (R v4.2.1) [25], and significance was assessed with Type-II Wald χ^2^ tests using the Anova() function in the “car” package.

Next, we analysed the movement and pausing patterns of termites across species. The succession of movements and pauses is a primary component of movement patterns [20, 26, 27], which is highly variable among species [21]. First, we obtained the distributions of the step lengths and found that they were bimodal for all five species, with the two peaks corresponding to individuals pausing and moving (Fig. S1). To define the threshold for pausing/moving behavior for each species, we estimated the kernel density of all step lengths and identified two modes: a near-zero pause mode (maximum within 0–0.5 mm) and a movement mode (largest local maximum at > 0.5 mm). We defined the threshold as the minimum density (valley) between these two modes; steps < threshold were labeled pause, and steps ≧ threshold were labeled move. Using this threshold, we converted trajectories into sequences of moving and pausing behaviours and computed the durations of each behaviour for all individuals. We removed the step lengths with overly high values (larger than mean + 2SD for each individual), as they do not reflect termite walking behaviour and occurred when termites tried to climb up the glass wall and tumbled down, leading to large displacements of body center between successive frames.

We compared the proportion of pausing behaviour between nesting types using LMM, with nesting type as a fixed effect and species as a random effect within which colonies were nested (random intercept). For each individual, the proportion of pausing was logit-transformed after 0.01 was added to the observed proportion to prevent the values from converging to infinity [28]. We also compared the moving speed between one-piece nesters and foragers using similar LMMs. The moving speed was estimated for each individual by computing the mean of step lengths above the threshold value for movements.

We fitted stretched exponential distributions and truncated power law distributions to the durations of moving and pausing separately, using maximum likelihood methods [20, 26, 27]. Those probability distribution functions are described as follows. Stretched exponential: $P\left( x \right) = \lambda \beta {x^{\beta - 1}}exp\left\{ { - \lambda \left( {{x^\beta } - x_{min}^\beta } \right)} \right\}$, $(x \ge {x_{min}}, \lambda > 0, 0 < \beta \le 1)$ and truncated power law: $P\left( x \right) = {{\left( {\mu - 1} \right){x^{ - \mu }}} \over {x_{min}^{1 - \mu } - x_{max}^{1 - \mu }}} , ({x_{min}} \le x \le {x_{max}}, \mu > 1)$. We chose these two distributions because they are biologically interpretable and represent alternative forms of constraints on movement. The truncated power law reflects heavy-tailed, scale-free movement patterns, with a cut-off imposed by physical or physiological limits [29]. In contrast, the stretched exponential reflects stronger constraints than scale-free behavior, in which the probability of long bouts decays rapidly and long movements become increasingly rare [26]. Our objective was therefore not to identify the single best-fitting distribution in a purely statistical sense, but to test which type of constraint (weak vs. strong) better explains the observed movement patterns in relation to termite nesting ecology. We also fit simple exponential and power-law distributions for comparison. Because bout durations were recorded at discrete frame intervals (0.2 s) due to the camera’s fixed frame rate, we used discrete cumulative distribution functions (CDFs) to obtain unbiased parameter estimates, following the approach described in previous studies [20, 26, 27]. Parameters were estimated by maximum likelihood using custom R scripts, and support for each model was evaluated using Akaike information criterion (AIC) weights. To quantify absolute goodness-of-fit, we also report Kolmogorov–Smirnov (KS) statistics (*D*) for each model fit. In addition to these two main models, we also fitted a simple exponential and a non-truncated power law as reference benchmarks, using the same bin-based maximum likelihood procedure. These additional comparisons, summarized in Supplementary Table S2, confirmed that truncated power law or stretched exponential distributions consistently provided the best fit across all species and behaviours.

Finally, we further investigated the interspecific variations in time spent in the inner/outer region (Fig. 4). We divided the experimental arena into two distinct regions: the inner and outer regions. The inner region was defined as the region inside a centered circle (Ø = 140/√2 mm), while the outer region was the rest of the arena. This definition of the inner and outer regions implies that the surface area of both regions was identical. The proportion of time spent in each zone was logit-transformed and compared between nesting types using LMMs with nesting type as a fixed effect and colonies nested within species as random intercepts. To further characterize how termites traversed the arena center, we identified “inner bouts,” defined as continuous runs within the inner region lasting at least 1.0 s (≥5 consecutive frames at 5 fps). For each bout, we calculated four metrics: (i) duration (seconds), (ii) path length (mm), (iii) chord length between entry and exit points (mm), and (iv) straightness index (SI = chord length/path length). These bout-level measures were averaged per individual. Individuals that never entered the inner region were excluded from the analysis. The resulting per-individual averages were then analyzed using LMMs with foraging status as a fixed effect and species and colonies nested within species as random intercepts. All analyses were performed with R v4.2.1 (https://www.R-project.org/). The R scripts used in this study are available as supplementary material.

### Ethical note

All data collection and experiments in this study were conducted in accordance with institutional guidelines on the ethical treatment of invertebrates. Care was taken to minimize stress to the collected colonies; for example, colonies were kept under stable laboratory conditions (20°C) with adequate food and moisture. No termites died or were subjected to procedures involving significant harm during the observations. Following the completion of the experiments, all colonies were maintained in the laboratory. We do not believe that our procedures had any long-term adverse effects on the health or survival of the colonies.

## Results

The traveled distance of termite workers was highly variable among species (Fig. 2A–B). After accounting for variation in body size by standardising traveled distances with body length, we found that one-piece nesting termites (*N. sugioi* and *Z. nevadensis*) traveled shorter distances than foragers (*H. sjostedti*, *C. formosanus*, and *O. formosanus*) (Fig. 2C; LMM, χ^2^_1_ = 7.51, *p* = 0.006). Note that Fig. 2B plots traveled distance against body length; thus, spatial proximity in this panel reflects body-size similarity rather than behavioural similarity. For example, *H. sjostedti* appears close to one-piece nesters due to comparable body size, yet its traveled distance clearly falls within the foraging range (Fig. 2C). The traveled distance was not largely affected by the original colonies (Fig. S2), as the variance decomposition of the LMM indicated that most between-individual variation was attributable to species identity (57.5%), with only a small contribution of colony (3.8%) and the remainder residual (38.7%).

**Fig. 3:**
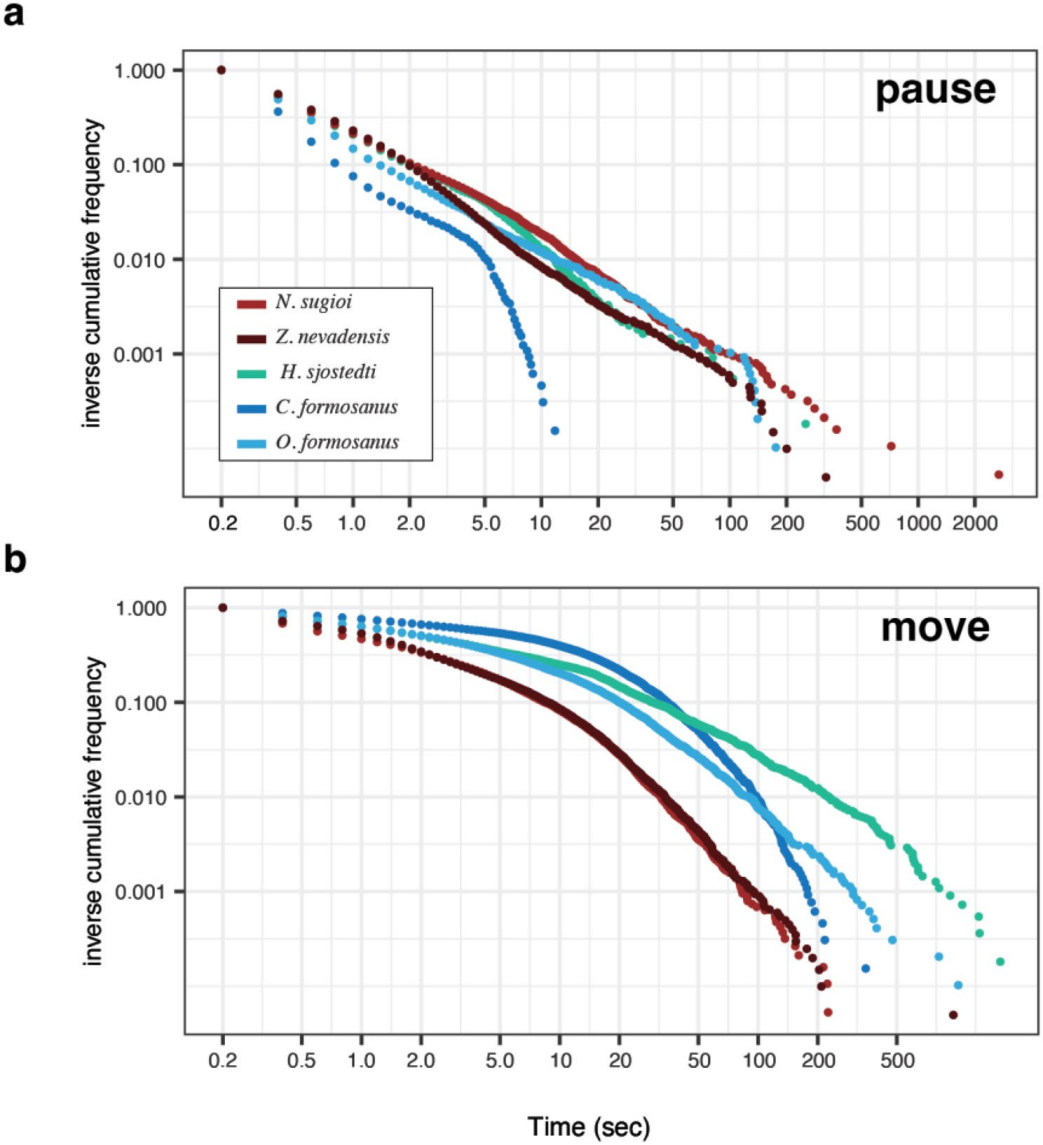
Comparison of pause- and movement-bout duration patterns across termite species. Curves show the inverse cumulative distribution function (ICDF), defined as the proportion of bouts with duration greater than or equal to a given value, plotted on log–log axes. This representation highlights the long-tail properties of the distributions and facilitates comparison of their shapes. Bout durations were pooled across all individuals within each species and analysed separately for (**a**) pause and (**b**) movement bouts. Numbers of bouts analysed (pause/move) were: *Z. nevadensis* (20,149/20,166), *H. sjostedti* (5,498/5,521), *N. sugioi* (18,880/18,894), *C. formosanus* (6,487/6,511), *O. formosanus* (9,738/9,760). For each group, four distribution models were fitted (truncated power law, stretched exponential, exponential, and power law; statistical supports are provided in table S2)

We found consistent differences in moving and pausing patterns between one-piece nesters and foragers. Model fitting showed that, with the exception of *C. formosanus* pause durations (best described by a truncated power law), both movement and pause bout durations across all species were better captured by stretched exponential distributions than by truncated power laws (Table S2, Fig. S1). Thus, both one-piece nesting and foraging species exhibited constrained bout-duration profiles, rather than scale-free behaviors. However, the shape of the distribution of movement and pausing durations illustrated the different patterns between foragers and one-piece nesters. The patterns of pausing durations were qualitatively similar across species based on the shape of inverse cumulative distribution functions (ICDFs) (Fig. 3), except for *C. formosanus*. On the other hand, the patterns of movement durations were distinct between foragers and one-piece nesters. One-piece nesters typically exhibited short movement bouts, with > 90% of movements terminating within 10 s. By contrast, foragers displayed more gradual declines in their ICDFs in shorter periods, indicating that locomotion was more likely to persist once movement was initiated. For example, the proportion of movements that lasted longer than 100 seconds was markedly higher for foragers than for one-piece nesters. Divergence among foraging species became particularly pronounced at longer timescales ( > 20 s), reflecting species-specific behaviors.

**Fig. 4:**
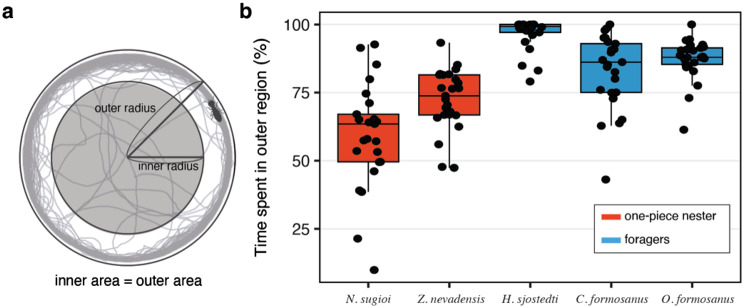
Comparison of time spent in the outer region. (**a**) Schematic illustrating the definition of the outer region. (**b**) Percentage of total amount of time spent in the outer region during the 60-minute observation. All species spent more time in the outer region than in the center of the arena. Foraging species spent significantly more time in the outer region than one-piece nesters (LMM, Type-II Wald χ^2^ test on logit-transformed proportions with random effects of species/Colony: χ^2^_1_ = 5.21, *p* = 0.022)

All species spent more time in the outer region than in the center of the arena (Fig. 4). Especially, several individuals of foragers never entered the inner region during the observation period (*C. formosanus*: 1/25, *O. formosanus*: 1/25, *H. sjostedti*: 7/25). This outer preference was stronger in foragers, which occupied the outer region significantly longer than one-piece nesters (LMM, χ^2^ ₁ = 5.21, *p* = 0.022). Variance decomposition indicated that species identity explained 25.0% of the variance in time spent in the outer region, colony 6.8%, and residuals 68.2%. Movement patterns within the inner region differed markedly between foragers and one-piece nesters (Fig. S4). Individuals of one-piece nesting species spent longer periods and covered greater path lengths inside the inner circle compared with foragers (LMM, mean duration: χ^2^ ₁ = 16.5, *p* < 0.001; mean path length: χ^2^ ₁ = 17.8, *p* < 0.001). In contrast, the net displacement between entry and exit points (chord length) was greater in foragers (χ^2^ ₁ = 7.54, *p* = 0.007). As a result, straightness indices were significantly higher in foragers, indicating a greater tendency to traverse the inner zone along more direct routes (χ^2^ ₁ = 73.3, *p* < 0.001).

## Discussion

In this study, we investigated the relationship between nesting types and worker movement patterns in five termite species and found that the movement patterns of termites reflect their self-built living environments. One-piece nesters have a small territory limited to their nesting wood piece, while foragers, which include multiple-piece and separate nesters, have a broader range as they forage outside their nests. Accordingly, we found that one-piece nesters traveled significantly shorter distances than multiple-piece and separate nesters (Fig. 2). Our results show a link between nesting type and termite movement patterns, suggesting that individual animal movements are products of the adaptation to their living environments.

Temporal patterns of locomotion revealed systematic differences between nesting types. Although stretched exponential models generally provided the best fit, the ICDFs showed that foragers sustained movement for longer once initiated, reflected in their shallower initial slopes relative to one-piece nesters (Fig. 3). This pattern is consistent with their ecology. Foragers regularly leave their nests to explore external environments, where prolonged movements may facilitate efficient search and resource exploitation. One-piece nesters, restricted to a single wood piece, typically exhibited brief bouts that terminated quickly. Pausing patterns, on the other hand, were broadly consistent between one-piece nesters and foragers, with a notable exception in *C. formosanus* (Fig. 3). For this species, AIC favored the truncated power law for pause durations, yet the goodness of fit is very poor for any distributions (Fig. S1, Table S2). The pauses of *C. formosanus* were extremely brief and infrequent, resulting in only a small peak near zero in the step-length distribution (Fig. S1). Taken together, these results indicate that termite locomotion patterns can be highly variable across species, even within foragers.

Beyond the movement activities, foragers and one-piece nesters also differed in spatial preferences. Both groups spent more time near the wall than in the central area, but this tendency was stronger in foragers. In fact, several foraging termites spent all the observational period near the wall (Fig. 4). Such differences in spatial preference likely stem from variation in positive thigmotaxis, or wall-following behavior [30–37]. During our observations, termites often maintained antennal contact with the wall while walking, similar to observations in cockroaches. In cockroaches, tactile stimulations from the wall help maintain their position near the wall and elicit exploratory activity [33, 38, 39]. In addition to the wall-following behavior, movement patterns within the inner area also contributed to the differences in spatial preferences. Foragers tended to cross the center area more directly, spending less time in the inner area; one-piece nesters exhibited longer and more tortuous paths, spending more time. Therefore, movement patterns of one-piece nesters and foragers are qualitatively distinct, highlighting the phenotypic diversity in innate behavior linked to nesting strategies.

Finally, our results suggest that the correlation between termite nesting strategies and movement patterns may serve as a proxy to classify termite nesting types. For example, *H. sjostedti* was initially described as a one-piece nester but was later confirmed to be a multiple-piece nester according to the field observation [16]. Our movement observations strengthen this field observation by showing that *H. sjostedti* exhibited movement patterns distinct from other one-piece nesters. Similar patterns may also be observed in other social insects with different foraging strategies [40]. Note that the five studied species did not only differ in their nesting strategies but also in colony size, social structures, and caste systems [41], affecting various social behaviours [42–45]. Expanding this research with additional termite species will be required to test the robustness of these findings.

## Conclusion

Our study is the first to suggest an association between termite nesting types and movement patterns. Our observations were performed under controlled laboratory conditions, which are largely different from termite natural living environments. The choice of setting, such as the shape and size of experimental arenas, could also influence the movement patterns [46]. In addition, we observed individuals one by one, which likely influences the behaviour of these social insects living in groups [47]. Despite the artificiality of our setup, our standardised approach allows direct comparisons of movement patterns among termite species, revealing a link with nesting biology. By highlighting the relationship between nesting biology and movement patterns, this study provides an example of the contribution of living environments to shaping innate behaviours. Future studies across broader taxa will contribute to our understanding of the role of movement in the evolution of social complexity.

## Electronic supplementary material

Below is the link to the electronic supplementary material.

Supplementary Material 1

## Data Availability

Data that support the findings of this study are available in Zenodo: 10.5281/zenodo.17603016.
