# Supplementary material for: Nesting strategy reflects individual worker movement in termites
Source: Mov Ecol. 2025 Dec 29;13:88. doi: 10.1186/s40462-025-00612-y (PMC12751460; doi:10.1186/s40462-025-00612-y)
Supplement: Supplementary file 1 — Supplementary Material 1 [file 40462_2025_612_MOESM1_ESM.pdf]

Supplemental materials for
"Nest complexity reflects individual worker movement of termites"

Kensei Kikuchi[1], **Thomas Bourguignon**[1], **Nobuaki Mizumoto**[1,2*],
1: Okinawa Institute of Science & Technology Graduate University, Onna-son, Okinawa, 904-0495, Japan
2: Department of Entomology & Plant Pathology, Auburn University, Auburn, AL, 36849, USA
*. Correspondence: Nobuaki Mizumoto; nzm0095@auburn.edu

E-mail:
KK: Kensei.Kikuchi@oist.jp ; NM: nzm0095@auburn.edu
ORCID: KK: 0009-0008-7509-7966 NM: 0000-0002-6731-8684

The file includes
Fig. S1. Histogram of the length of displacements between successive frames (0.2 s) and inverse cumulative distribution function (ICDF) of the duration of moves and pause for each termite species.
Fig. S2. Per-colony total distance (body lengths) by species.
Fig. S3. Per-colony time spent in the inner region (%) by species.
Fig. S4. Movement geometry during inner region bouts by species.
Table S1. Summary of movement metrics of termite workers across different nesting types.
Table S2. Results of model fitting to moving and pausing time data.

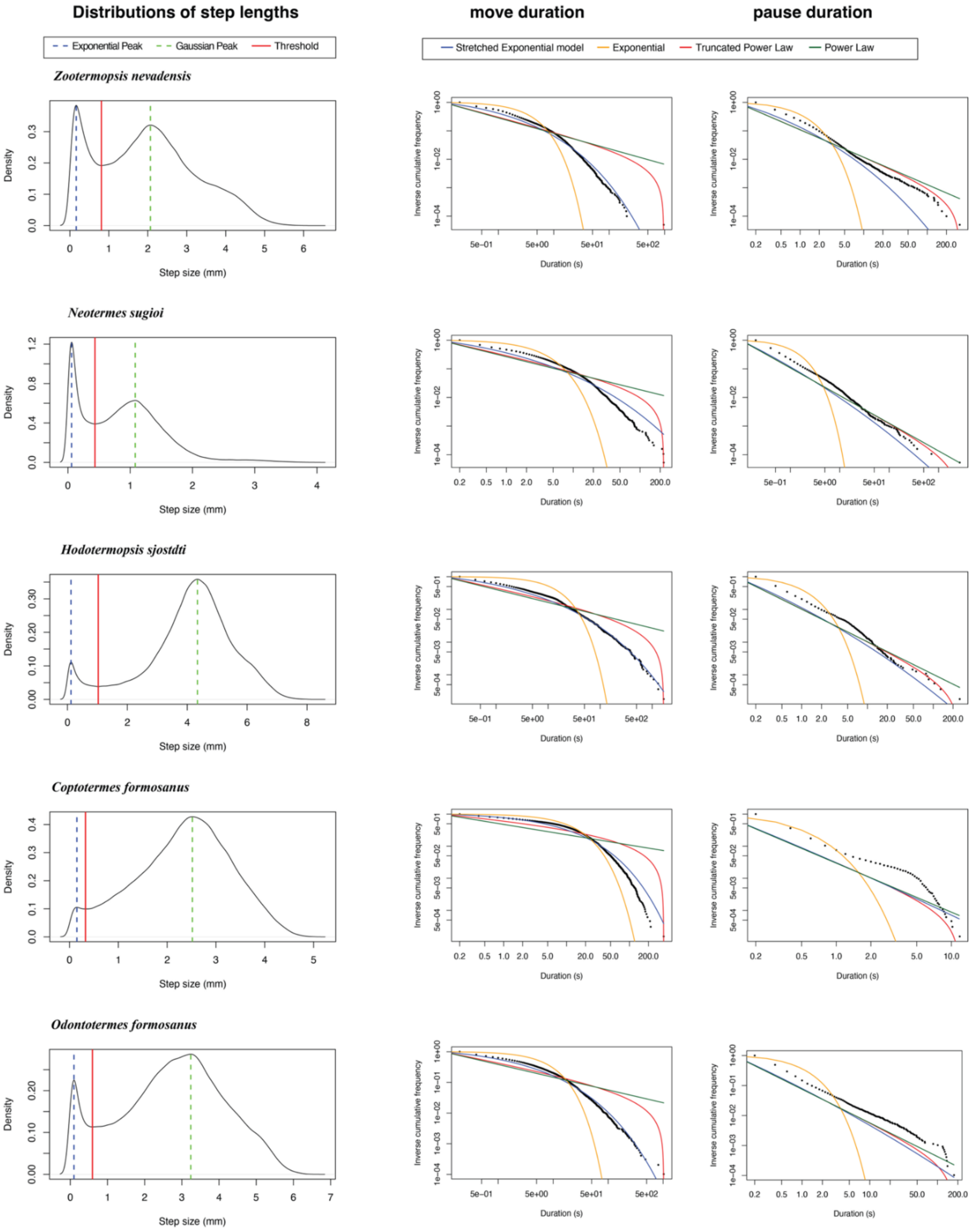

Figure. S1. Distributions of step lengths (displacements between successive frames; 0.2 s) and inverse cumulative distribution functions (ICDFs) of movement and pause durations for each termite species. In the histograms, the two peaks correspond to the near-zero pause mode and the movement mode. The green dotted line indicates the second (movement) peak of the fitted Gaussian distribution, the blue dotted line indicates the pause peak of the fitted exponential distribution, and the red solid line represents the threshold used to distinguish pauses from movements. In the ICDF plots, the red curve shows the fitted truncated power-law model, the blue curve shows the fitted stretched exponential model, the yellow curve shows the exponential, and the green curve shows the power law.

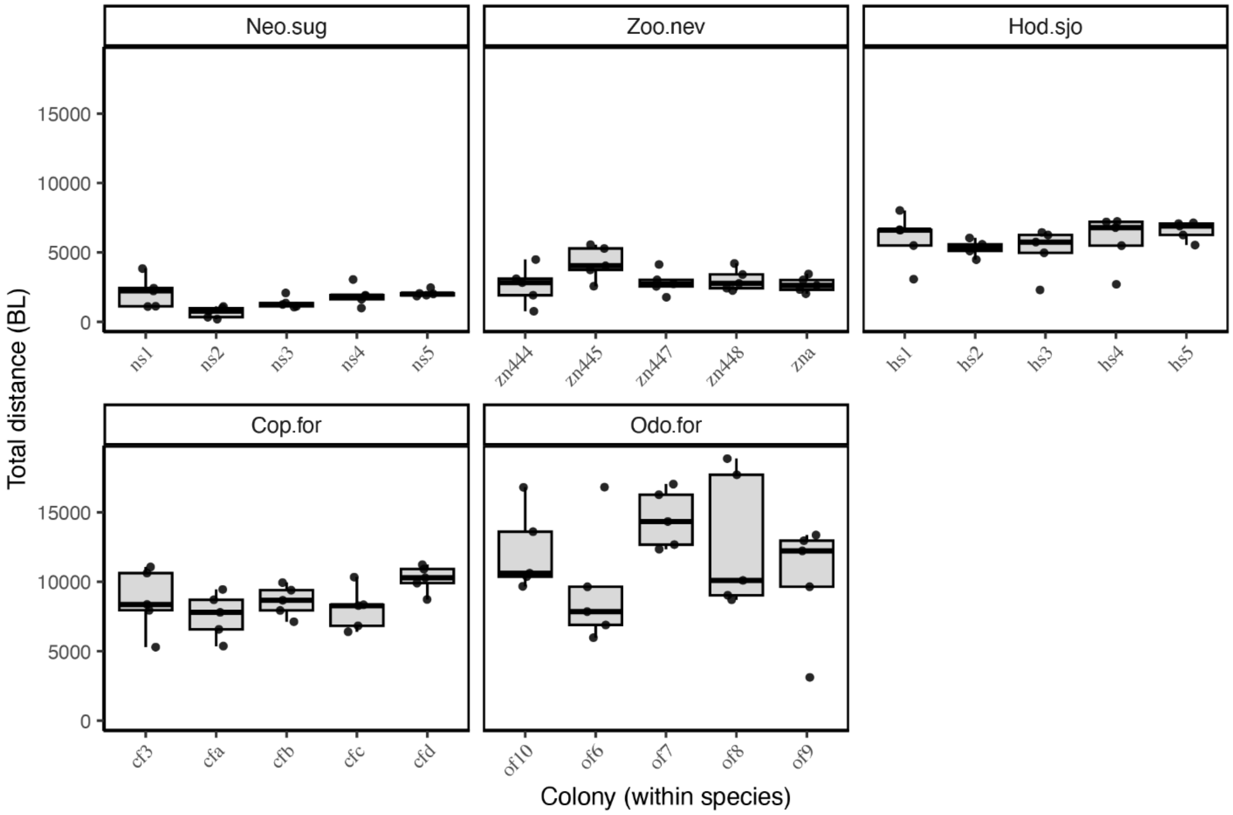

Figure. S2. Comparison of total traveled distance across colonies. Boxplots show median, interquartile range (IQR) box, and 1.5×IQR whiskers; points are individual workers (jittered). These plots illustrate modest among-colony variation within species relative to between-species differences.

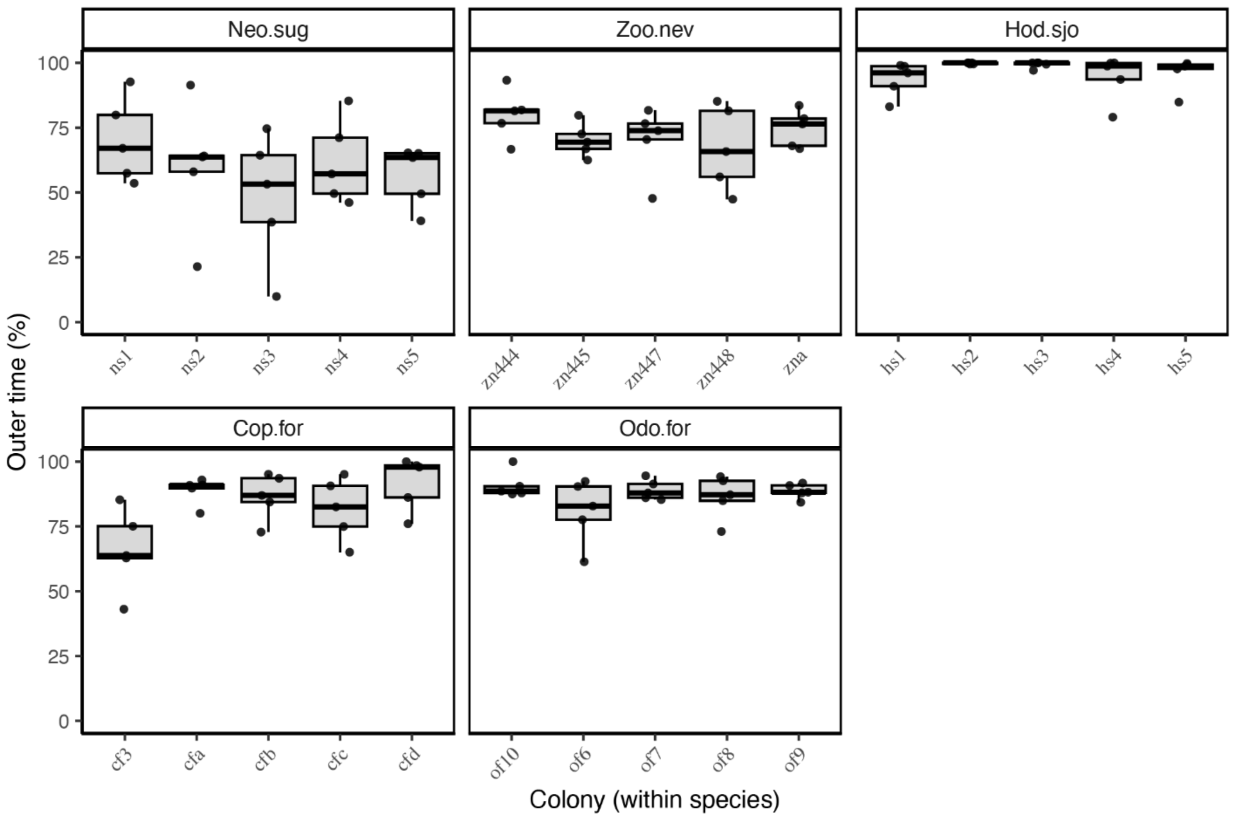

Figure. S3. Comparison of time spent in the outer region (%) across colonies. The outer region is defined as the equal-area central circle (radius = dish radius/√2). Per-colony distributions overlap broadly within species, consistent with small colony-level variance estimated by the LMMs.

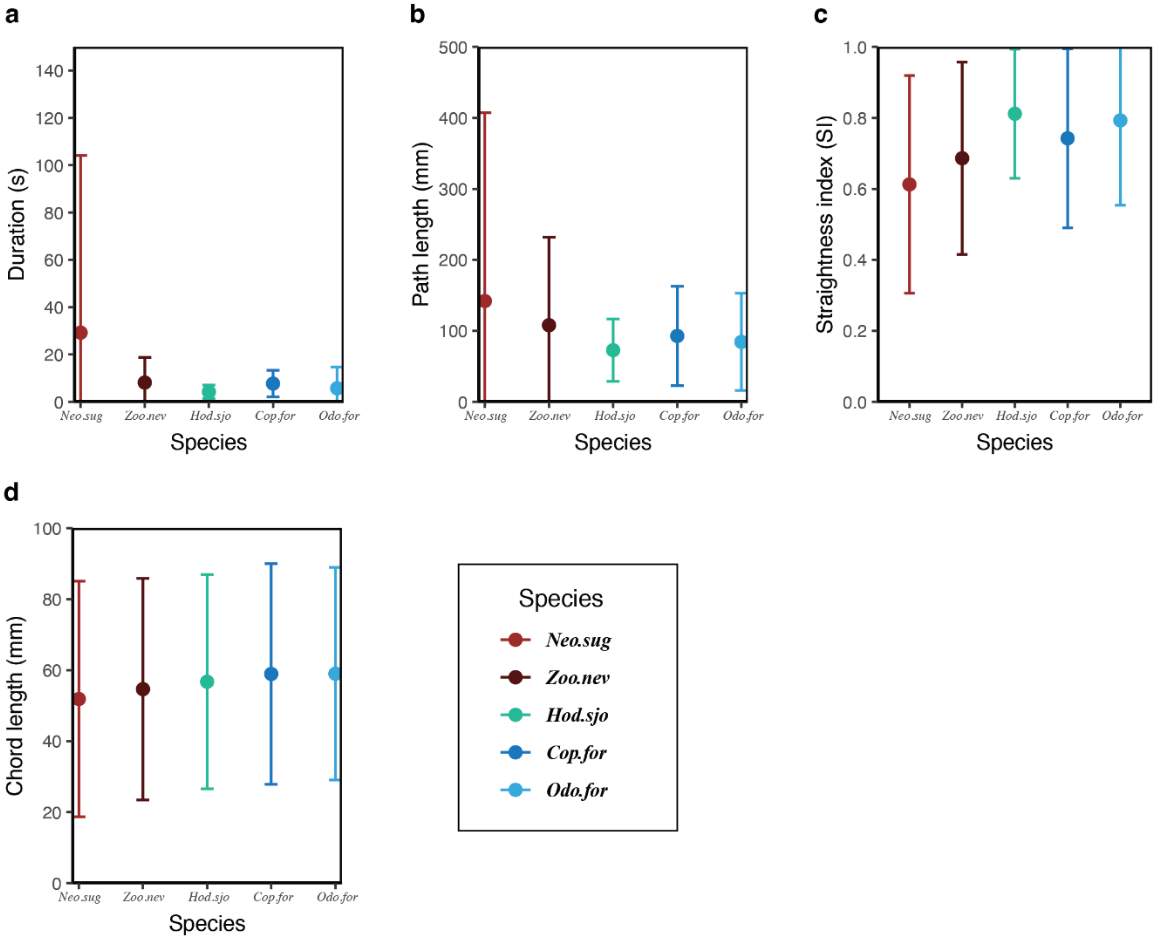

Figure S4. Movement geometry during inner region bouts by species (mean ± SD). (a) Duration of inner bouts (s). (b)Path length travelled within inner bouts (mm). (c) Straightness index (SI= Chord/ Path) bounded in [0,1]. (d) Chord length between bout entry and exit points (mm). Points show species means; error bars denote ± SD across bouts pooled over individuals within species.

Table S1. Summary of movement metrics of termite workers across different nesting types.

**Species**	**Nesting**	**Foragers**	**Speed (mm)**	**Speed (BL)**
C. formosanus	Separate	Yes	12.32	2.55
H. sjostedti	Multiple-piece	Yes	20.81	1.66
N. sugioi	One-piece	No	5.36	0.57
O. formosanus	Separate	Yes	15.38	3.79
Z. nevadensis	One-piece	No	10.86	0.95

The table includes species names, nesting type, moving speed, and speed standardised by body length (BL).

Table S2. Results of model fitting to moving and pausing time data.

Species	*Z. nevadensis*		*N. sugioi*		*H. sjostedti*		*C. formosanus*		*O. formosanus*	
Behavior	move	pause	move	pause	move	pause	move	pause	move	pause
Bouts	20166	20149	18894	18880	5521	5498	6511	6487	9760	9738
Min data	0.2	0.2	0.2	0.2	0.2	0.2	0.2	0.2	0.2	0.2
Max data	965	324	225.4	2680	1664.2	252.6	349	11.8	1021.6	175.4
μ(TP)	1.51	1.96	1.52	1.97	1.33	1.99	1.14	2.51	1.35	2.12
μ(PL)	1.54	1.96	1.58	1.97	1.39	2.00	1.32	2.53	1.42	2.12
β(SE)	0.321	0.198	0.244	0.0654	0.257	0.0887	0.522	0.0324	0.343	0.0486
λ(SE)	1.69	5.51	2.38	15.3	1.31	11.8	0.375	48.5	1.04	23.9
λ(EXP)	0.303	1.10	0.321	0.822	0.0664	1.02	0.0757	2.87	0.124	1.13
AIC_TP	139185	85157	123970	79667	49100	22582	66236	17120	83077	35585
AIC_SE	136704	84636	123219	79604	48568	22561	64621	17147	81485	35580
AIC_PL	139641	85190	124763	79671	49515	22592	68412	17147	83814	35595
AIC_EXP	153464	101447	141546	107373	58761	28547	67595	20357	91703	48517
wTP	0	0	0	0	0	0	0	0.999	0	0.0797
wSE	1	1	1	1	1	0.999	1	0	1	0.920
wPL	0	0	0	0	0	0	0	0	0	0
wEXP	0	0	0	0	0	0	0	0	0	0
Best	SE	SE	SE	SE	SE	SE	SE	TP	SE	SE
KS_TP_D	0.302	0.486	0.309	0.488	0.214	0.496	0.165	0.648	0.224	0.540
KS_SE_D	0.182	0.401	0.221	0.458	0.132	0.458	0.0800	0.641	0.119	0.519
KS_PL_D	0.314	0.488	0.330	0.489	0.239	0.498	0.285	0.653	0.250	0.541
KS_EXP_D	0.277	0.340	0.345	0.425	0.406	0.391	0.215	0.387	0.306	0.419

Model-fitting results for movement and pause bout duration distributions. Each column corresponds to a termite species and each row reports a model parameter or goodness-of-fit value (estimated separately for movement and pause data). "Min data" and "Max data" denote the minimum and maximum observed bout durations. For the truncated power law (TP), μ(TP) is the power-law exponent. For the pure power law (PL), μ(PL) is the corresponding exponent without truncation. For the stretched exponential (SE), β is the stretching parameter ($\beta < 1$ indicates heavier tails than an exponential, $\beta = 1$ corresponds to a pure exponential) and λ(SE) is the rate parameter. For the exponential model (EXP), λ(EXP) is the rate parameter. Model comparison is summarized using the Akaike Information Criterion (AIC_TP, AIC_SE, AIC_PL, AIC_EXP) and Akaike weights (wTP, wSE, wPL, wEXP), which quantify the relative support for each model (i.e. the probability of being the best model in the candidate set). "Best" indicates the model with the highest Akaike weight ($w > 0.5$). Goodness-of-fit is evaluated using Kolmogorov–Smirnov statistics (KS_D), with smaller values indicating closer correspondence between the fitted and empirical distributions. "Bouts" is the total number of observed bouts included in the fit.Model.
